# Primary clostridium difficile infection in patients with ulcerative colitis: Case report and literature review

**DOI:** 10.1097/MD.0000000000036693

**Published:** 2024-02-09

**Authors:** Xizhuang Gao, Huihui Zhou, Zongjing Hu, Quanyi Wang, Yun Chen, Fengqin Zh, Guangxi Zhou

**Affiliations:** a Department of Clinical Medicine, Jining Medical University, Jining, Shandong, P.R. China; b Department of Gastroenterology, Affiliated Hospital of Jining Medical University, Jining Medical University, Jining, Shandong, P.R. China; c Pathology Department, Affiliated Hospital of Jining Medical University, Jining Medical University, Jining, Shandong, P.R. China.

**Keywords:** clostridium difficile, infliximab, ulcerative colitis

## Abstract

**Rationale:**

Inflammatory bowel disease (IBD), including Crohn disease (CD) and ulcerative colitis (UC), is a chronic immune-mediated disorder characterized by inflammation of the gastrointestinal tract. Patients with IBD are susceptible to various complications, including the coexistence of Clostridioides difficile infection (CDI). The incidence of IBD combined with difficile infection is higher in patients with compromised immune function, which can lead to increased mortality.

**Patient concerns:**

A 43-year-old male presented with recurrent episodes of mucus and bloody stools persisting for more than a month without any identifiable triggering factors. Initially, the stool consistency was normal, but it progressively shifted to a loose and watery texture, with up to 8 occurrences daily.

**Diagnoses:**

This case underscores the diagnosis of severe UC through colonoscopy and colonic biopsy, along with the supplementary identification of a positive result for Clostridioides difficile in the fecal sample.

**Interventions:**

The patient initiated infliximab therapy alongside a full vancomycin course, demonstrating the potential effectiveness of this intervention in managing early-stage ulcerative colitis with concurrent Clostridioides difficile infection.

**Outcomes:**

Following the completion of a full vancomycin course, the patient initiated infliximab therapy. The patient was free from significant discomfort, exhibited no fever, and had no mucopurulent bloody stools. A follow-up blood test indicated reduced inflammatory markers compared to the preoperative period, and the stools were normal.

**Lessons:**

We illustrate the potential effectiveness of this medication by presenting an in-depth case report of a patient with early-stage UC. The report outlines the patient inclusion of infliximab to better manage UC inflammation alongside an adjunct vancomycin regimen, given the ineffectiveness of mesalazine therapy and the concurrent presence of Clostridium difficile infection. This case prompts consideration of therapeutic approaches for complex UC and contributes to advancing both research and clinical practice. Nonetheless, we should remain attentive to the variations and potential risks unique to each patient in order to formulate personalized treatment strategies.

## 1. Introduction

Inflammatory bowel disease (IBD) is a group of chronic diseases that includes Ulcerative Colitis (UC) and Crohn Disease (CD). Although the precise mechanisms of these diseases are not fully understood, research suggests that genetics, immune system abnormalities, environmental factors, and gut microbiota may play crucial roles in their development. Abnormal immune responses in IBD patients result in persistent inflammation of the intestinal mucosa, which can potentially affect the entire gastrointestinal tract. UC, a subtype of IBD, predominantly affects the colon and rectum. Typical symptoms include abdominal pain, diarrhea, rectal bleeding, anemia, and fatigue. In UC, inflammation usually spreads continuously, starting from the rectum and extending upwards to various segments of the colon. Treatment strategies aim to reduce inflammation, alleviate symptoms, manage the disease, and enhance the quality of life^[1]^

Clostridium difficile infection (CDI) is caused by Clostridium, and the bacterium produces toxins that can damage the intestinal mucosa, resulting in inflammation and diarrhea. Symptoms of CDI include severe diarrhea, fever, abdominal pain, and nausea.^[2]^ Infections often occur in patients undergoing prolonged antibiotic therapy or immunosuppressive treatment, which happen to be common treatment approaches for UC patients. CDI can potentially lead to severe complications, including intestinal necrosis and systemic infection, especially in individuals already affected by IBD, potentially worsening the condition and complicating treatment.^[3]^

We have previously determined that patients with UC are at a significantly higher risk of acquiring CDI and its related complications.^[3]^ Research suggests that individuals with IBD are about 5 times more likely to develop CDI compared to those without IBD. Moreover, the prognosis for IBD patients affected by CDI is significantly worse, which includes prolonged hospitalizations, higher rates of colon resection, and increased mortality rates. Moreover, the prognosis for IBD patients affected by CDI is significantly worse, which includes prolonged hospitalizations, higher rates of colon resection, and increased mortality rates.^[4]^ Additionally, IBD patients exhibit a higher prevalence of asymptomatic carriage of Clostridium difficile than the general population.^[5]^

Management of CDI in the context of IBD poses a challenge, as it often shows a limited response to antibiotics. In this report, we present a case of a patient with UC complicated by CDI.

## 2. Case presentation

A 43-year-old male presented with recurrent episodes of mucus and bloody stools for over a month without any apparent triggering factors. Initially, the stool consistency was formed but gradually transitioned into a loose and watery texture, occurring up to 8 times daily. The stools were mixed with a small amount of fresh blood and pus, and the patient experienced lower abdominal pain, bloating, discomfort, urgency, and a feeling of incomplete evacuation. The patient was admitted to the local hospital, where an electronic colonoscopy was performed, suggesting UC. Despite receiving treatment with acid suppression, gastric protection, modulation of gut microbiota, and anti-inflammatory therapy, the patient reported unsatisfactory results.

The patient is currently experiencing a fever and requires further treatment. The patient initially sought medical attention at our hospital (Jining Medical College Affiliated Hospital) on September 10, 2021. The patient denies any history of chronic conditions such as hypertension or diabetes, previous surgeries, medication use, or food allergies. Physical examination revealed mild tenderness upon gentle palpation of the entire abdomen, without other positive signs such as rebound tenderness.

**Figure 1.**
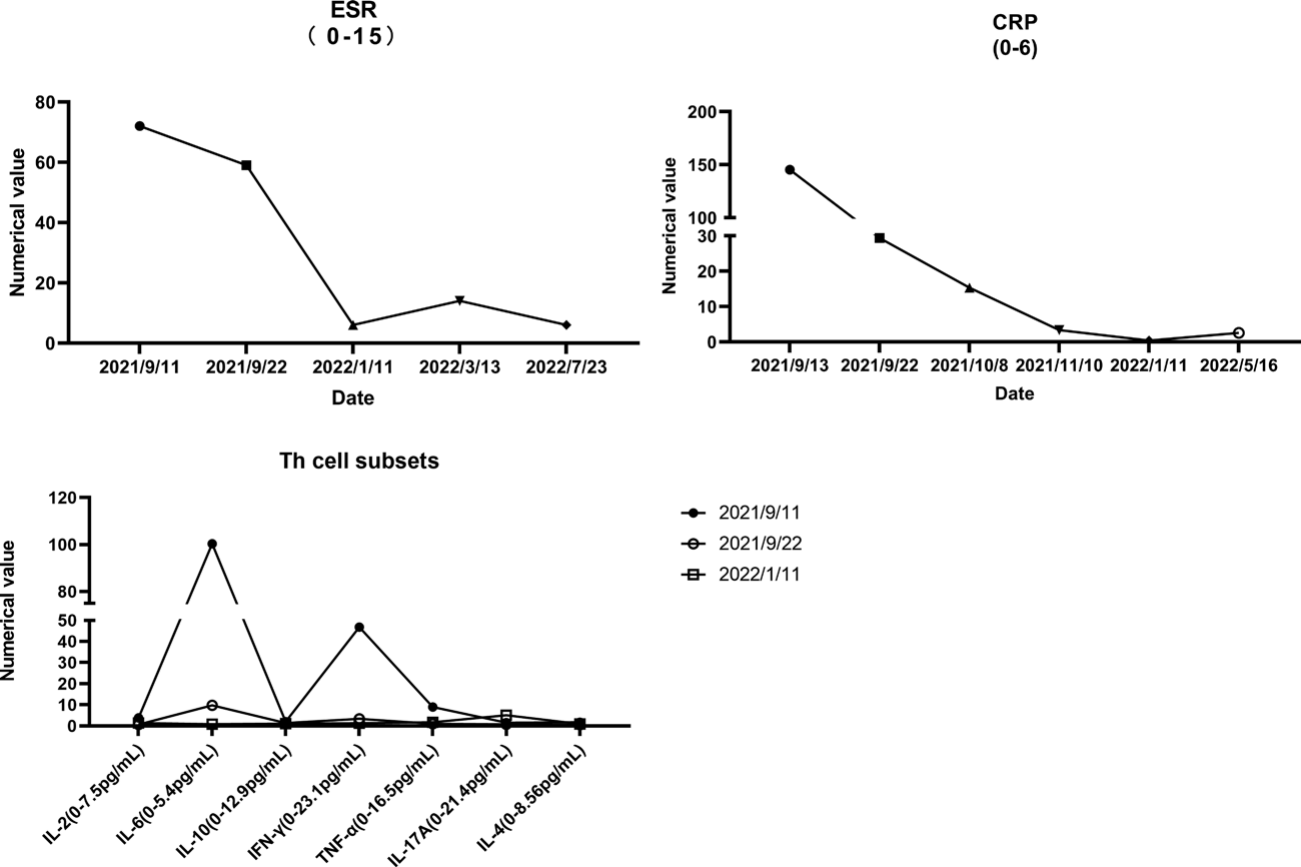
Erythrocyte sedimentation rate, Th cell subsets, and C-reactive protein results. CRP = C-reaction protein, ESR = erythrocyte sedimentation rate.

**Figure 2.**
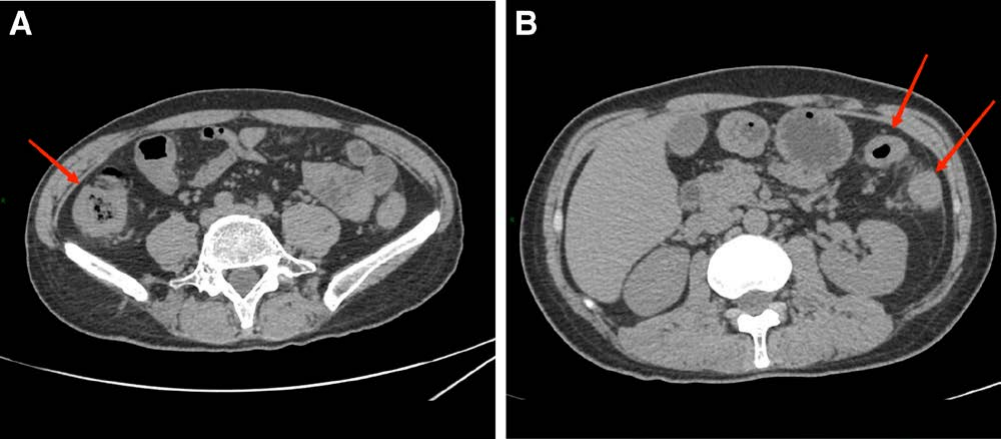
CT image of the abdomen: Images show thickening of the walls and surrounding infiltration in the cecum ascending colon, transverse colon, and descending colon(arrows), suggestive of inflammatory lesions.

**Figure 3.**
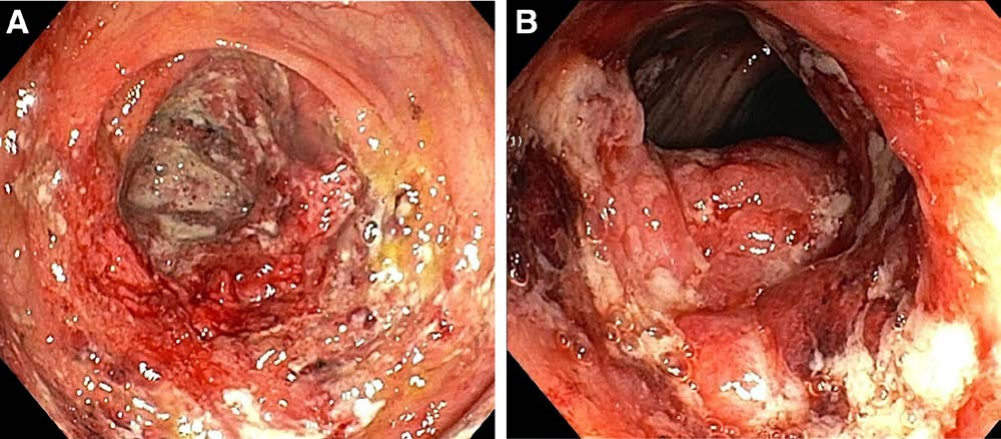
Colonoscopy images: Images show (A) Ileoceca and (B) transverse colon blurred vascular texture, scattered mucosal erosions and ulcers, and a little white moss at the base.

**Figure 4.**
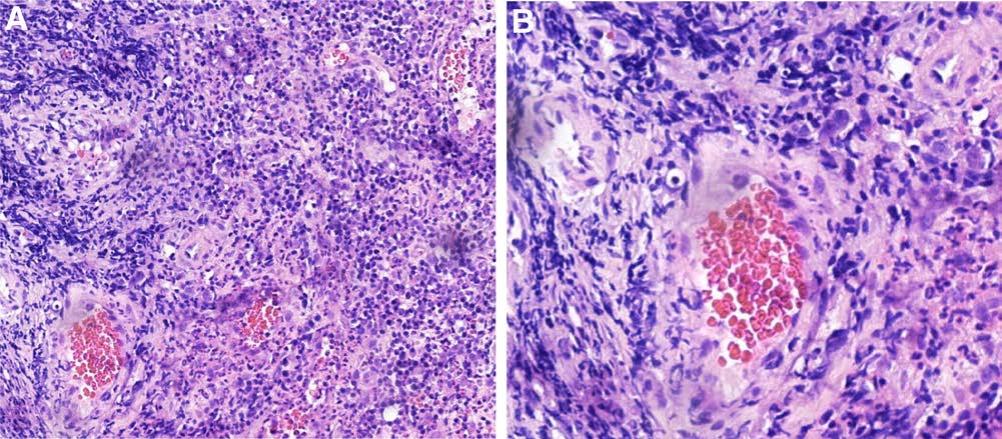
The image shows acute and chronic inflammation of the (ascending colon) mucosa with eosinophilic granulocytic infiltration and lymphoid hyperplasia with microabscesses and granulation tissue formation. (A×200, B×400).

On September 11^th^, the patient developed a fever with a temperature of 38.5°C and reported 5 episodes of mucus and bloody stools. A preliminary diagnosis of multiple sclerosis was initially made based on observations at the local hospital. Furthermore, pertinent ancillary investigations were conducted, including the assessment of hematocrit and T-cell subpopulations (Fig. 1). Therefore, with symptomatic and supportive therapy, treatment was initiated with omeprazole, mesalamine, kaolin, and pectin suspension. However, the fever symptoms persisted. On September 12^th^, an abdominal computed tomography scan was performed (Fig. 2), which indicated thickening of the walls and surrounding infiltration in the cecum, ascending colon, transverse colon, and descending colon, suggestive of inflammatory lesions. The patient had 9 episodes of loose mucus and bloody stools on the same day. Enhanced testing for C. difficile was advised due to the presence of severe intestinal lesions as indicated by previous colonoscopy results. Furthermore, the patient had a recorded temperature of 37.3°C and elevated CRP levels (Fig. 1), which suggested the presence of infection on September 13^th^. On September 14^th^, toxigenic Clostridioides difficile was detected in the patient stool culture. A follow-up Colonoscopy was performed on September 15^th^ (Fig. 3), revealing colonic congestion, edema, blurred vascular pattern, severe ulceration, erosion, and white coating in localized areas. On September 18^th^, a histopathological examination of the colonic mucosa biopsy showed infiltration of eosinophils, increased lymphoid tissue, formation of abscesses, and granulation tissue (Fig. 4). Throughout this period, the patient fever symptoms persisted without improvement.

Based on the clinical presentation, colonoscopy imaging, pathological findings, and computed tomography scans, the most likely diagnosis is UC with concurrent CDI. The patient has been experiencing persistent fever and 5 episodes of mucus and bloody stools. Initially, the patient was administered oral mesalamine at a dose of 2g twice daily, but there was no significant improvement in their condition. A combined treatment approach was decided upon after consultation with an infectious disease specialist. Oral vancomycin at a dose of 0.25g twice daily was prescribed for a treatment duration of 10 to 14 days.

In addition, given the severity of the intestinal lesions and the potential for bacterial infection, while waiting for the vancomycin regimen to be completed, it was decided to add infliximab to control further the inflammation associated with UC. With infliximab, the patient did not experience any significant discomfort, nor did she have fever, mucus, or bloody stools.

**Figure 5.**
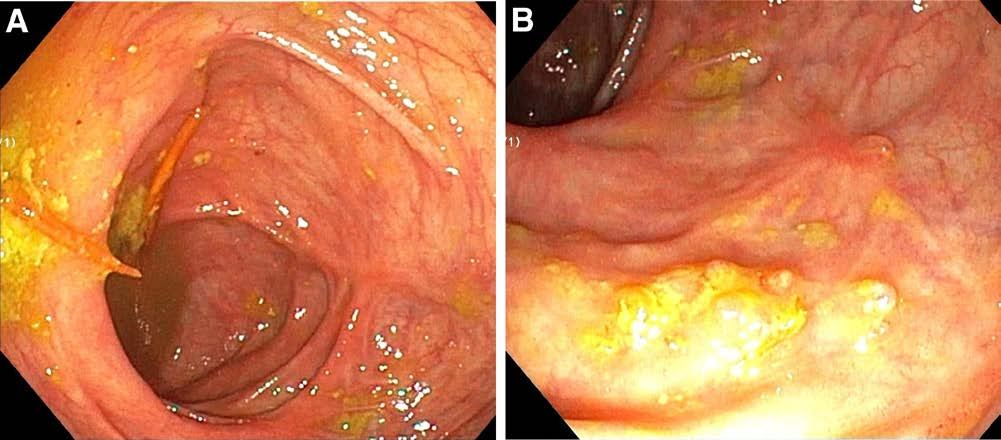
Colonoscopic image of January 11, 2022: the image shows localized mucosal edema with blurred vascular texture in (A) the ileocecal region and (B) the transverse colon; the mucosa at the opening of the ileocecal region is smooth, and there are scattered scarring changes in the transverse colon.

**Figure 6.**
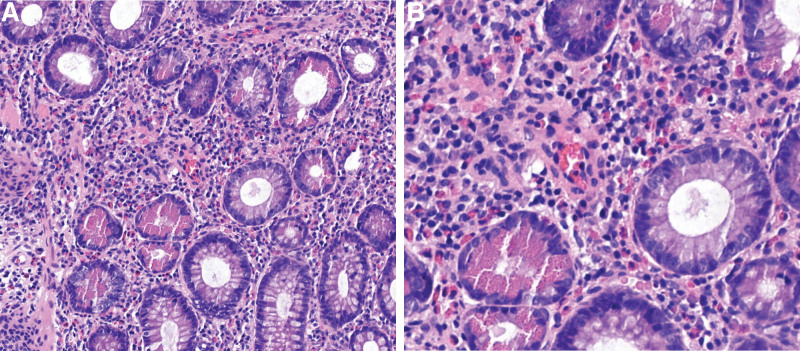
The January 11,2022 image shows an eosinophilic infiltrate in the (ascending colon). (A×200, B×400).

**Figure 7.**
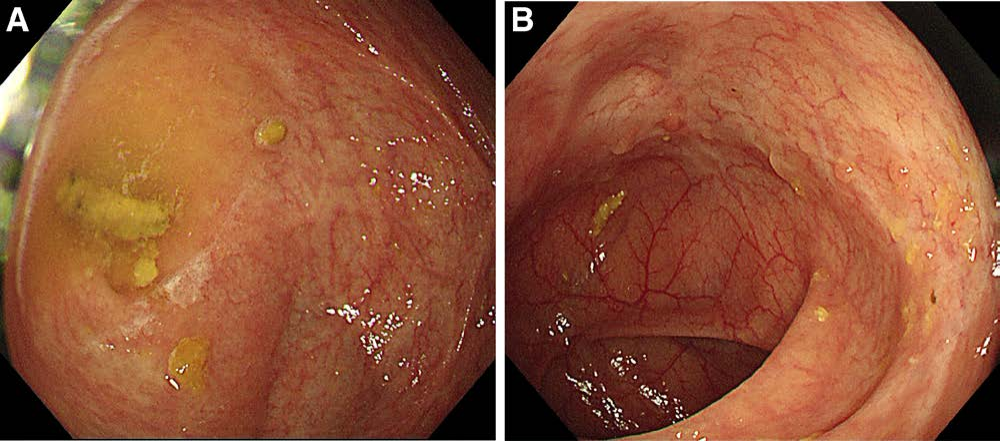
Colonoscopic image of January 7, 2023: The image shows localized slight congestion of the mucosa in (A) the ileocecal region and (B) the transverse colon, with no abnormality in the ileocecal region and scattered scar-like changes in the colon.

**Figure 8.**
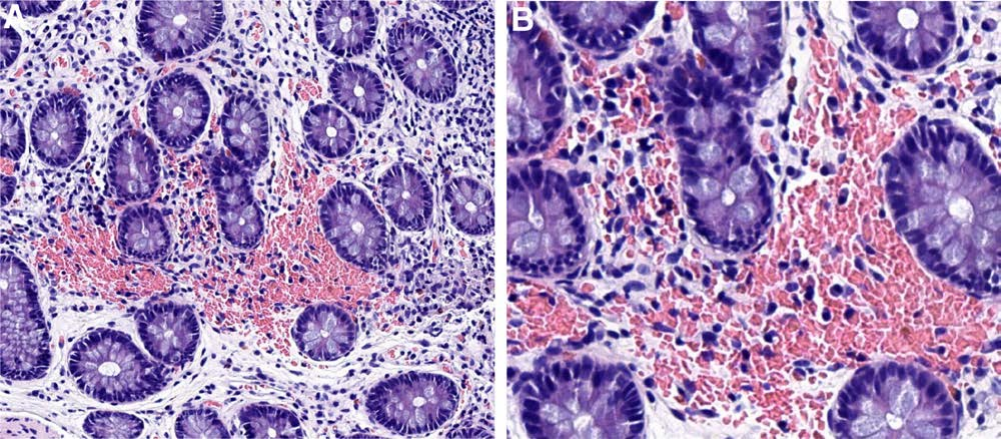
The image from January 7, 2023 shows an eosinophilic infiltrate (ascending colon). (A×200, B×400).

Following that, the patient received infliximab treatment on both October 7th and November 7th at our hospital. Subsequent blood tests during follow-up revealed notable decreases in ESR, CRP, and TH cell subsets when compared to their respective pretreatment levels (Fig. 1). Furthermore, the e-colonoscopy and pathology reports from January 11, 2022, and January 5, 2023, demonstrated a substantial enhancement in the condition of the colon when compared to the earlier evaluation (Figs. 5–8). The patient bowel movements returned to normal, and the venous thromboembolism risk assessment score remained at 0.

## 3. Discussion

IBD is a collective term encompassing a group of chronic IBD, among which UC and CD are included UC is one of these conditions, typically characterized by inflammation and ulceration that primarily affect the inner lining of the colon. Common symptoms of UC include diarrhea, abdominal pain, and blood in the stool. UC is characterized, among other aspects, by chronic inflammation and ulceration affecting the colon inner lining.^[6]^ The pathophysiology of this disease is complex, and its etiology remains largely unknown. However, dysbiosis of the intestinal microbiota is widely recognized as an essential factor in the development and progression of IBD.^[7]^ Clostridium difficile is a Gram-positive, spore-forming, anaerobic bacillus commonly distributed in the human intestinal tract. The bacterium often triggers an infection after antibiotic treatment because antibiotics disrupt the normal intestinal flora, allowing C. difficile to take advantage of it. Typical symptoms include diarrhea, abdominal pain and fever. It serves as an opportunistic pathogen and is associated with a range of diseases and infections.^[8]^

Indeed, it has been documented that the global incidence of CDI is rising. Notably, individuals with IBD who acquire CDI tend to experience a more severe disease progression. This includes a heightened risk of adverse outcomes, increased incidence, and elevated mortality rates. The co-occurrence of CDI and IBD presents a significant clinical challenge and underscores the importance of prompt and effective management strategies for these patients.^[9,10]^

The 2013 guidelines from the American College of Gastroenterology (ACG) recommend that all IBD patients who experience disease flare-ups or develop new-onset diarrhea should be tested for CDI. Diagnosis of CDI infection usually relies on specific laboratory tests, such as detecting clostridial toxins in a patient stool sample. This recommendation is because the symptoms of CDI can mimic those of an IBD flare-up, and the presence of CDI can exacerbate the disease course and outcomes in individuals with IBD. Hence, it is essential to promptly and accurately diagnose CDI to manage and treat IBD patients8 effectively. The PCR detection of toxin-producing genes is currently considered the preferred method for CDI diagnosis. It offers rapid testing with high sensitivity and specificity.^[11]^ Enzyme immunoassay is a fast, cost-effective method for detecting toxins in fecal samples. However, its sensitivity and specificity may vary.^[12]^ Therefore, the testing should be performed only on unformed fecal samples to reduce the false-positive rate.

The ACG experts recommend considering IBD as an indicator of severity for CDI. They suggest using vancomycin as the initial antibiotic treatment, followed by managing immunosuppression for persistent infection. However, treatment options may be limited for CDI comorbid with UC because certain antibiotics may negatively affect patients with UC. The ACG 2013 guidelines propose empirical treatment for CDI and IBD in patients with severe colitis while awaiting difficult-to-culture results. They advise continuing immunomodulatory therapy during CDI treatment. Initiating corticosteroids or anti-tumor necrosis factor therapy within the first 72 hours8 is not recommended. Close monitoring of patients for worsening symptoms of CDI and potential complications, such as toxic megacolon or perforation, is essential.

Therefore, combining both diseases must be considered for treating UC combined with CDI. Often, the CDI infection must first be controlled to reduce the symptoms of diarrhea and sickness. Then, therapy of UC must be continued to control inflammation and signs in the colon. Critical steps in treating UC combined with CDI include choosing the appropriate antibiotic for CDI, usually vancomycin or metronidazole. Concurrent management of UC may require continued use of immunosuppressants or biologics, but this needs to be done under the supervision of a physician. Closely monitor the patient symptoms and immune system status to adjust the treatment plan. Follow infection control measures to prevent transmission of CDI.

Use of the biologic agent infliximab: Infliximab is a monoclonal antibody that targets tumor necrosis factor-alpha and has been widely used to treat IBD such as UC and CD.^[13]^ The use of infliximab: Infliximab is a monoclonal antibody that targets tumor necrosis factor-alpha and has been widely used for the treatment of IBD such as UC and CD.^[14]^

This treatment regimen primarily uses vancomycin to treat CDI and control bacterial infections. Infliximab, on the other hand, is used to treat UC to prevent the inflammatory response in the colon. This combination treatment strategy aims to manage UC and CDI to improve the patient overall health. Patients are subject to close medical supervision during treatment, including regular checkups for symptoms, laboratory tests, and immune system status.

Indeed, in clinical practice, treating IBD patients with concurrent CDI infection using antibiotics and immunosuppressive agents remains a challenge. Currently, there are no definitive guidelines, and the conclusions from relevant literature reports are often conflicting.^[15]^ A study comparing the effectiveness of combined immunomodulators and antibiotics versus using antibiotics alone for treating CDI in patients with IBD found that using multiple immunomodulators further increased the risk of adverse effects, while patients treated solely with antibiotics did not experience adverse outcomes.^[16]^ In another population-based study, no association was found between infliximab and an increased risk of developing CDI.^[17]^ The infusion of infliximab has been reported to lead to the resolution of recurrent CDI symptoms in a single case of UC.^[18]^

## 4. Conclusion

The detailed analysis of case reports in newly diagnosed UC patients reveals a successful vancomycin treatment approach. This treatment effectively controlled the infection and alleviated inflammatory symptoms in patients who did not respond to mesalamine therapy and developed CDI. The addition of infliximab to further manage inflammation while completing the vancomycin course demonstrated the potential efficacy of this medication. This case study provides valuable insights into the treatment strategies for complex UC and serves as a reference for further research and clinical practice. Nonetheless, it is crucial to consider individual variations and potential risks to develop personalized treatment plans for each patient.

## Author contributions

**Data curation:** Huihui Zhou.

**Investigation:** Zongjing Hu, Quanyi Wang.

**Methodology:** Yun Chen.

**Writing – original draft:** Xizhuang Gao.

**Writing – review & editing:** Fengqin Zh, Guangxi Zhou.
